# Supplementary material for: Pigeons exhibit low susceptibility and poor transmission capacity for H5N1 clade 2.3.4.4b high pathogenicity avian influenza virus
Source: J Gen Virol. 2025 Sep 17;106(9):002156. doi: 10.1099/jgv.0.002156 (PMC12444787; doi:10.1099/jgv.0.002156)
Supplement: Uncited Supplementary Material 1. [file jgv-106-02156-s001.pdf]

Table S1

Supplementary Table 1. Summary of observed clinical signs in pigeons and chickens, following initial pigeon inoculation with H5N1-AB.

	**Total counts of clinical sign occurrence**								
	**Low dose (10^2 EID$_{50}$)**			**Medium dose (10^4 EID$_{50}$)**			**High dose (10^6 EID$_{50}$)**		
**Clincial Sign**	**D0 Pigeon**	**R1 Pigeon**	**R1 Chicken**	**D0 Pigeon**	**R1 Pigeon**	**R1 Chicken**	**D0 Pigeon**	**R1 Pigeon**	**R1 Chicken**
Changes in huddling	0	**1**	0	0	0	0	0	0	0
Eyes closed	0	0	0	0	0	0	0	0	0
Conjunctivitis	0	0	0	0	0	0	0	0	0
Changes in body position	0	**1**	0	0	0	0	**1**	0	0
Oedema	0	0	0	0	0	0	0	0	0
Cyanosis of extremities	0	0	0	0	0	0	0	0	0
Lethargy	0	0	0	0	0	0	0	0	0
Lack of engagement with enrichment	0	0	0	0	0	0	0	0	0
Oronasal discharge	0	0	0	0	0	0	0	0	0
Diarrhoea	0	0	0	0	0	0	0	0	0
Perceived weight reduction	0	0	0	0	0	0	0	0	0
Loss of balance	0	0	0	0	0	0	0	0	0
Tremors	0	0	0	0	0	0	0	0	0
Torticollis	0	0	0	0	0	0	0	0	0
Seizure	0	0	0	0	0	0	0	0	0
Paralysis/inability to eat or drink	0	0	0	0	0	0	0	0	0
**Total**	0	**2**	0	0	0	0	**1**	0	0

D0, directly inoculated pigeons; R1, contact exposed pigeons or chickens.

Fig. S1

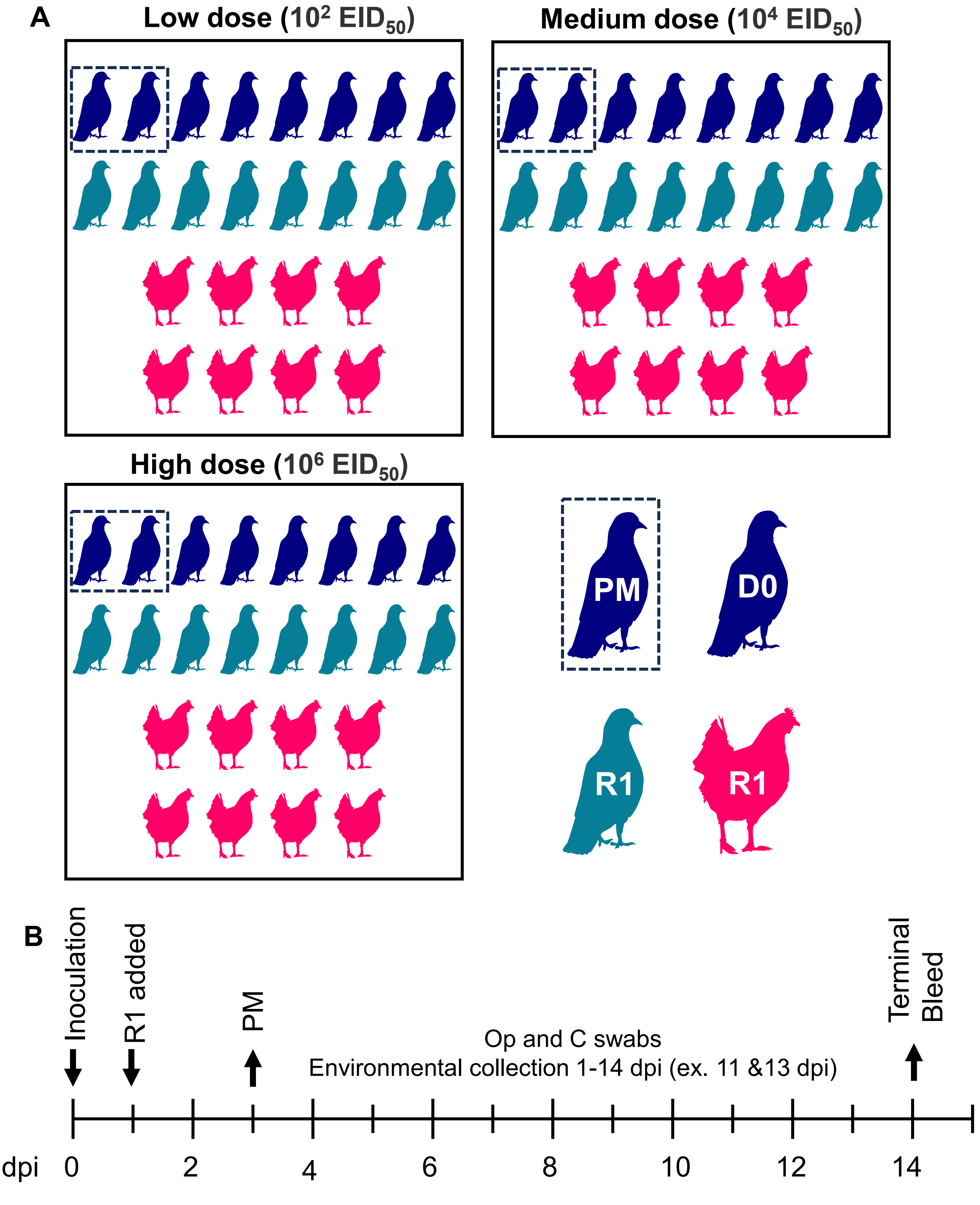

Fig. S1. Schematic diagram of the pigeon infection and transmission study design.
A. Schematic of the study design. Eight pigeons were directly infected (D0, blue) with low (10^2 EID$_{50}$), medium (10^4 EID$_{50}$) or high (10^6 EID$_{50}$) doses of H5N1-AB. Eight pigeons (teal) and eight chickens (pink) were co-housed (R1) with the D0 pigeons. Two D0 were culled for postmortem (PM) analysis (hashed box). **B.** timeline of the study showing key events with days post infection (dpi) shown.

Fig. S2

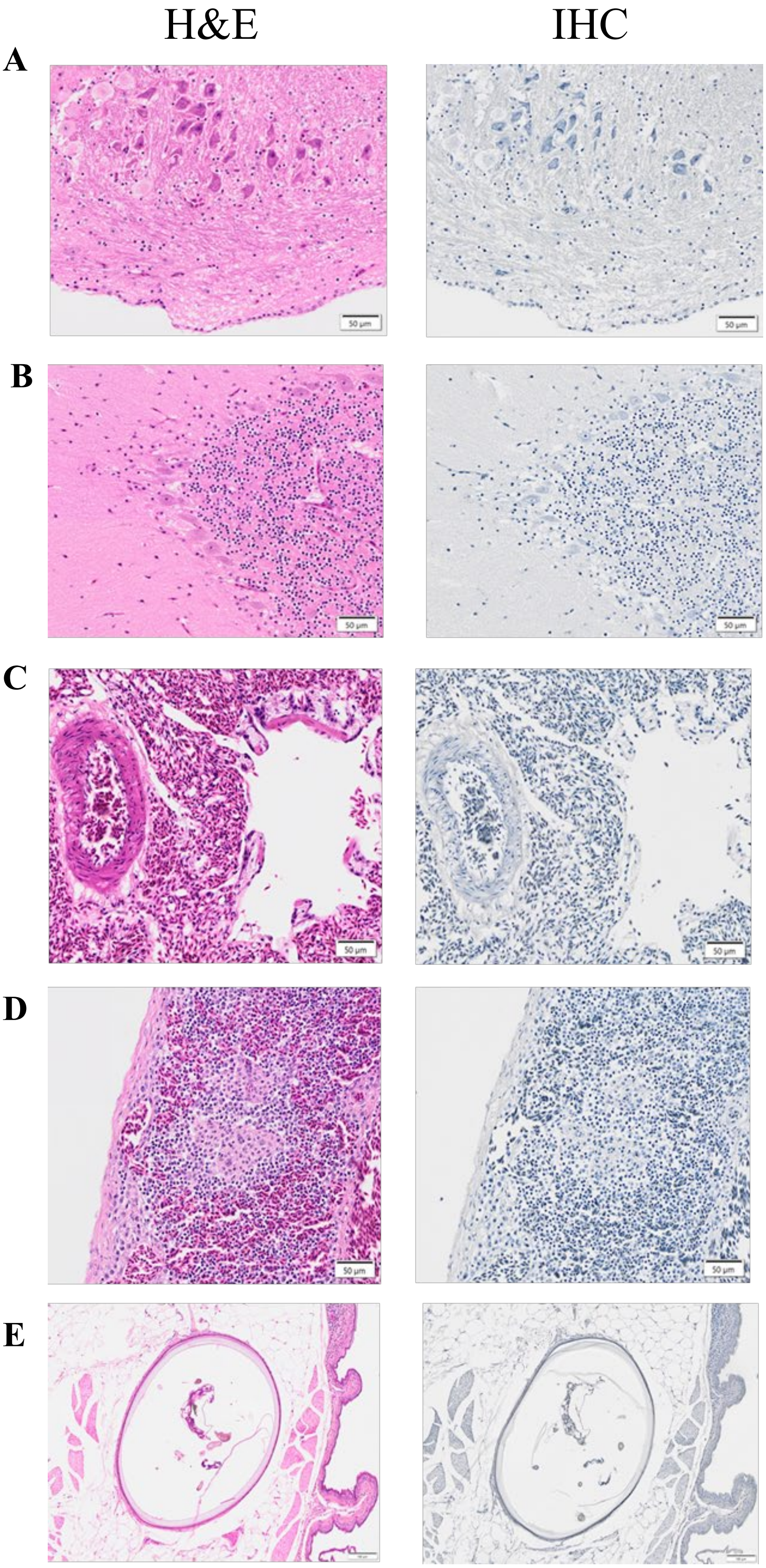

Fig. S2. Representative tissues sections used for pathological and influenza A viral nucleoprotein (NP) staining, following pigeon infection with H5N1-AB.

Representative images taken from scanned slides from pigeon #41 (3 dpi) and edited by Olympus Olyvia software. Tissue sections from the same location were stained with haematoxylin and eosin (H&E) (left) or used for virus-specific immunohistochemistry (IHC) (viral nucleoprotein would stain brown but was not detected in any tissue) (right). (**A**) Cerebrum (neurons, oligodendrocyte, microglial cells). (**B**) cerebellum (granule cell layer on right-hand side and molecular layer on the left with Purkinje neurons in-between). (**C**) spleen (red and white pulp). (**D**) lung showing major blood vessel (left-hand side) and parabronchus (right-hand side). (**E**) epidermis, dermis, subcutis and feather pulp.

Fig. S3

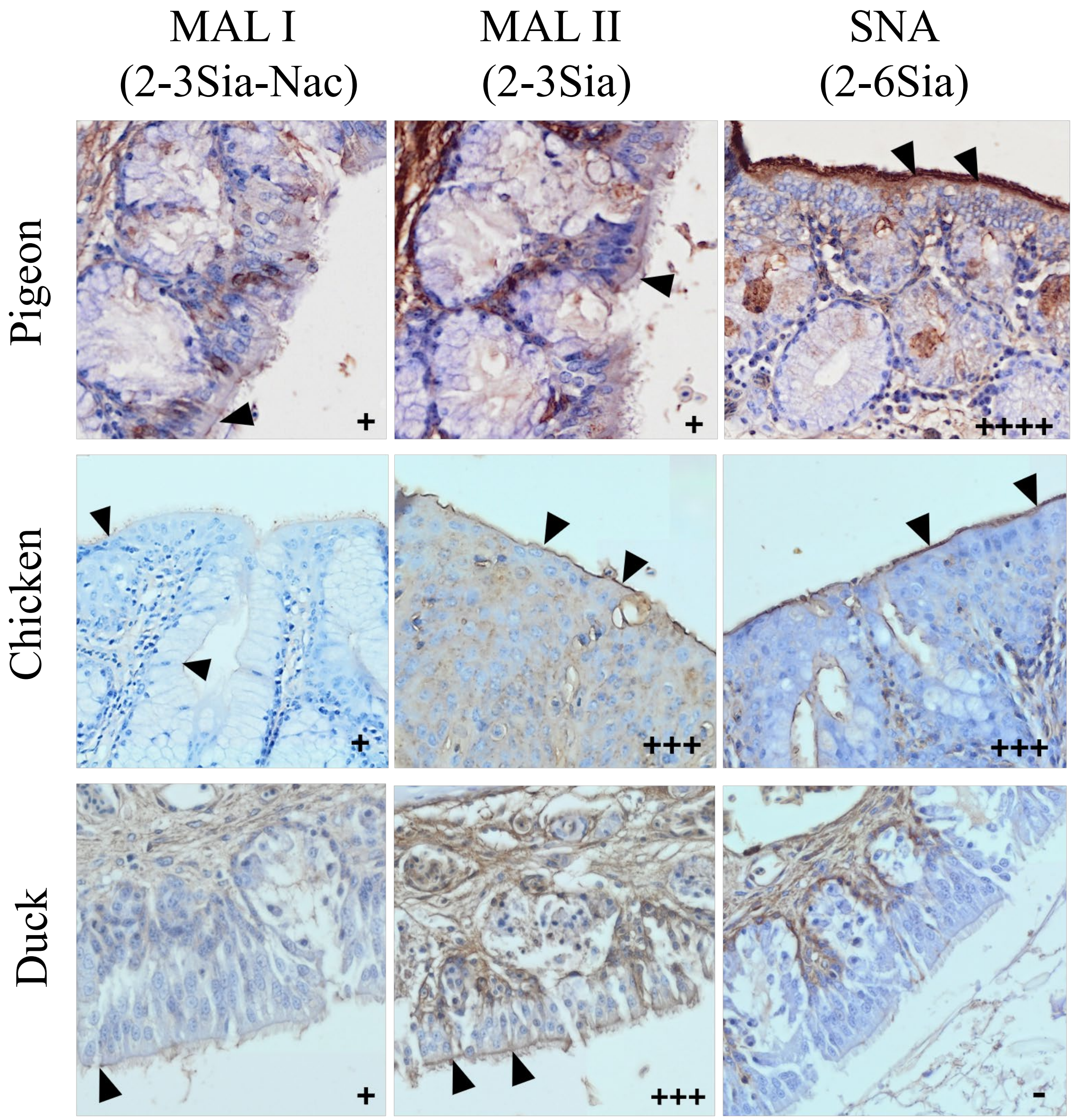

Fig. S3. Representative images of lectin histochemical labelling in the nasal turbinates of chickens, ducks and pigeons. Lectin staining demonstrates the distribution and relative abundance (from none (-) to abundant (++++)) of 2-3Sia and 2-6Sia receptors. The presence of receptors is demonstrated on the apical surface of the ciliated epithelial cells as brown line (black arrowheads). Data from these representative images across a range of additional respiratory tissues is summarised in Table 1.
